# Optimizing laparoscopic training efficacy by ‘deconstruction into key steps’: a randomized controlled trial with novice medical students

**DOI:** 10.1007/s00464-022-09408-2

**Published:** 2022-07-18

**Authors:** A. Widder, J. Backhaus, A. Wierlemann, I. Hering, S. Flemming, M. Hankir, C.-T. Germer, A. Wiegering, J. F. Lock, S. König, F. Seyfried

**Affiliations:** 1 Department of General-, Visceral-, Transplant-, Vascular- and Pediatric Surgery, Center of Operative Medicine (ZOM), University Hospital of Wuerzburg, Würzburg, Germany; 2 Institute of Medical Teaching and Medical Education Research, University Hospital of Wuerzburg, Würzburg, Germany

**Keywords:** Laparoscopic skills, Teaching methods, Deconstruction into key steps, Laparoscopic course

## Abstract

**Background:**

Simulator training is an effective way of acquiring laparoscopic skills but there remains a need to optimize teaching methods to accelerate learning. We evaluated the effect of the mental exercise ‘deconstruction into key steps’ (DIKS) on the time required to acquire laparoscopic skills.

**Methods:**

A randomized controlled trial with undergraduate medical students was implemented into a structured curricular laparoscopic training course. The intervention group (IG) was trained using the DIKS approach, while the control group (CG) underwent the standard course. Laparoscopic performance of all participants was video-recorded at baseline (t_0_), after the first session (t_1_) and after the second session (t_2_) nine days later. Two double-blinded raters assessed the videos. The Impact of potential covariates on performance (gender, age, prior laparoscopic experience, self-assessed motivation and self-assessed dexterity) was evaluated with a self-report questionnaire.

**Results:**

Both the IG (*n* = 58) and the CG (*n* = 68) improved their performance after each training session (*p* < 0.001) but with notable differences between sessions. Whereas the CG significantly improved their performance from t_0 _–t_1_ (*p* < 0.05), DIKS shortened practical exercise time by 58% so that the IG outperformed the CG from t_1 _-t_2_, (*p* < 0.05). High self-assessed motivation and dexterity associated with significantly better performance (*p* < 0.05). Male participants demonstrated significantly higher overall performance (*p* < 0.05).

**Conclusion:**

Mental exercises like DIKS can improve laparoscopic performance and shorten practice times. Given the limited exposure of surgical residents to simulator training, implementation of mental exercises like DIKS is highly recommended. Gender, self-assessed dexterity, and motivation all appreciably influence performance in laparoscopic training.

Minimally invasive access to the abdominal cavity (laparoscopy) is the standard approach for various procedures in visceral surgery [1, 2], since patients generally experience less postoperative pain, recover faster, and have improved perioperative morbidity and mortality [3–6]. As such, the laparoscopic approach is increasingly employed for more complex surgeries including oncological procedures [7–11].

To support the safe implementation of laparoscopic approaches, early and intensive training is required [12, 13]. However, working-hour restrictions and a high administrative workload severely limit the time young surgeons spend in the operating theatre. Adequate exposure time consequently poses a major challenge to surgical education along with the development of laparoscopic skills [14, 15].

Several studies have demonstrated the efficacy of virtual and non-virtual laparoscopic simulator training for the successful transfer of acquired skills into the clinical setting [16–18]. Structured laparoscopic simulator training not only shortens operating times, but also lowers costs for resources [13, 19]. However, while laparoscopic simulators exist at universities and teaching hospitals, young surgeons usually have limited access to them [20, 21].

For laparoscopic procedures such as cholecystectomy, the risks and complications can be mitigated when adhering to a standardized procedure. The evaluated mental exercise ‘deconstruction into key steps (DIKS)’ is a teaching method which meets these needs when learning basic laparoscopic skills [22]. Here, we implemented a prospective randomized controlled trial into a curricular laparoscopic skill course to investigate whether DIKS results in a similar improvement of performance compared to prolonged time to practice. The desired outcome was to identify a method that reduces the required exposure time to a laparoscopic simulator without impairing performance outcome [15]. Additionally, the impact of various covariates including gender, age, prior laparoscopic experience, self-assessed motivation and self-assessed dexterity on laparoscopic performance was evaluated. The purpose of this covariate analysis was to identify factors that enhance /diminish laparoscopic performance sui generis.

## Materials and methods

### Study design and course schedule

A structured, two-session laparoscopy course was constructed as an integral part of an obligatory two-week rotation in surgery ( 10th semester students) at the Julius-Maximilians-University of Würzburg, Germany. Informed consent was obtained from all participants. The study was approved by the local ethics committee (20,170,403 01).

At baseline, participants had to complete an online questionnaire (EvaSys®) containing demographic data, previous laparoscopic expertise, self-assessment of dexterity, as well as motivation. Participants were randomly assigned to either the intervention (IG) or the control group (CG).

Performance was measured at baseline (t_0_), at the end of the first session (t_1_), and nine days later (t_2_). Improvement of performance was determined as the difference between t_0 _-t_1_, t_1 _-t_2_ and t_0_-t_2_ using the normalized gain equation R. [23]. where the numerator is the difference between the pre-test and the post-test, and the denominator is the maximum achievable value minus the pre-test [24].

**Fig. 1:**
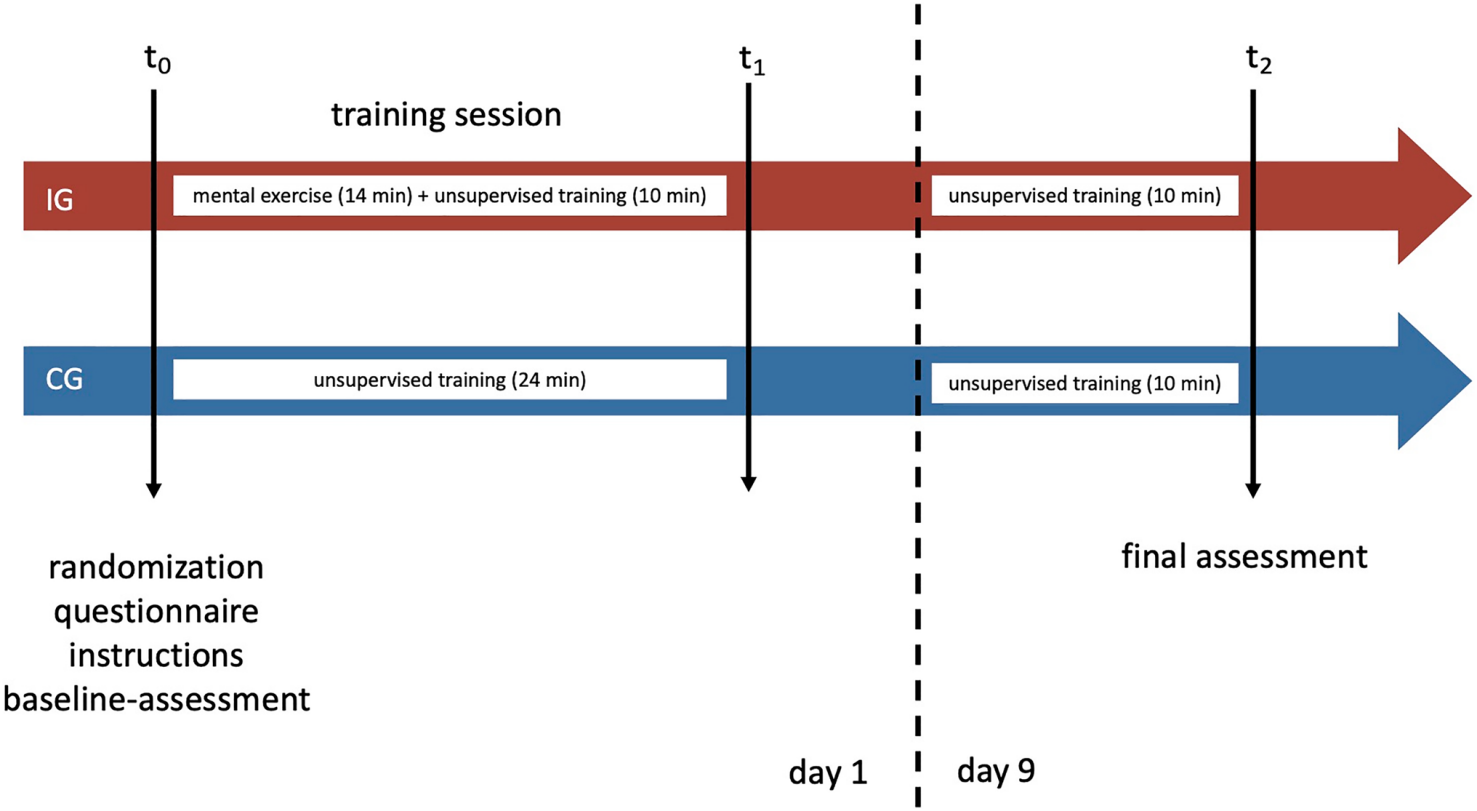
Study design and training for students in pairs

Students were trained in surgeon-camera assistant tandem pairs (Fig. 1) and standard introductory video tutorials were given to both the IG and the CG. Multimedia-based training is a valid method to teach complex motion sequences required for surgical procedures [25].

#### Intervention from t_0_-t_1_

The IG was instructed in using DIKS for 14 min, followed by unsupervised training for 10 min (5 min per participant in each pair). The CG spent the entire 24-min timeslot practicing laparoscopic skills using the simulator (12 min per participant in each pair). Time to practice for the IG was subsequently reduced by 58%. Participants were asked to make handwritten notes using their own words since this seems to aid with memorization [26–29]. Furthermore, the IG recorded their individual difficulties and corresponding solutions when performing the laparoscopic exercise.

#### Intervention from t_1_-t_2_

For IG and CG the second 10-min session (5 min per participant in each pair) was identical to the first session but was unsupervised and the IG was allowed to (re)examine their keysteps.

Performance of attending surgeons (*n* = 6) and surgical residents (*n* = 5) from the Department of Visceral Surgery, University Hospital Würzburg, Germany served as an internal validation.

### Training setup

For core training, the Berlin OP trainer (BOPT) was used [30]. The video unit comprised a 30° “Autoclave” lens and a “telecam PAL” camera module with a “telepac PAL” screen from Storz (KARL STORZ SE & Co. KG, Tuttlingen, Germany). “Click line overholts" with a length of 2 cm from Storz were used as instruments. Three Trocars (Endopath Xcel (Ethicon 12 mm); Ethicon J&J Medical Devices, Norderstedt, Germany) were used in a standardized way to access the BOPT’s cavity.

Laparoscopic training and performance were assessed using the laparoscopic training module "packing suitcases". This exercise was developed and validated as an integral teaching module of the “Lübecker Toolbox” (LBT) [31]. In order to enable integration of camera assistance and corresponding interactions, dimensions were modified (scale: 25.5 cm × 27.5 cm vs. LTB 12 cm x 12 cm). The aim of the task was to place all cups in two separate cases, depending on its color. For successful completion of the task, all cups had to be placed in the correct case within five minutes and sorted in an upright position.

In order to collect the students’ personal data and their self-assessments, a questionnaire was given using the survey software EvaSys (Copyright © 2021 EvaSys GmbH, Lüneburg, Germany). All participants were video-recorded throughout the three measurement points as surgeon and camera assistant,

### Performance rating

**Fig. 2:**
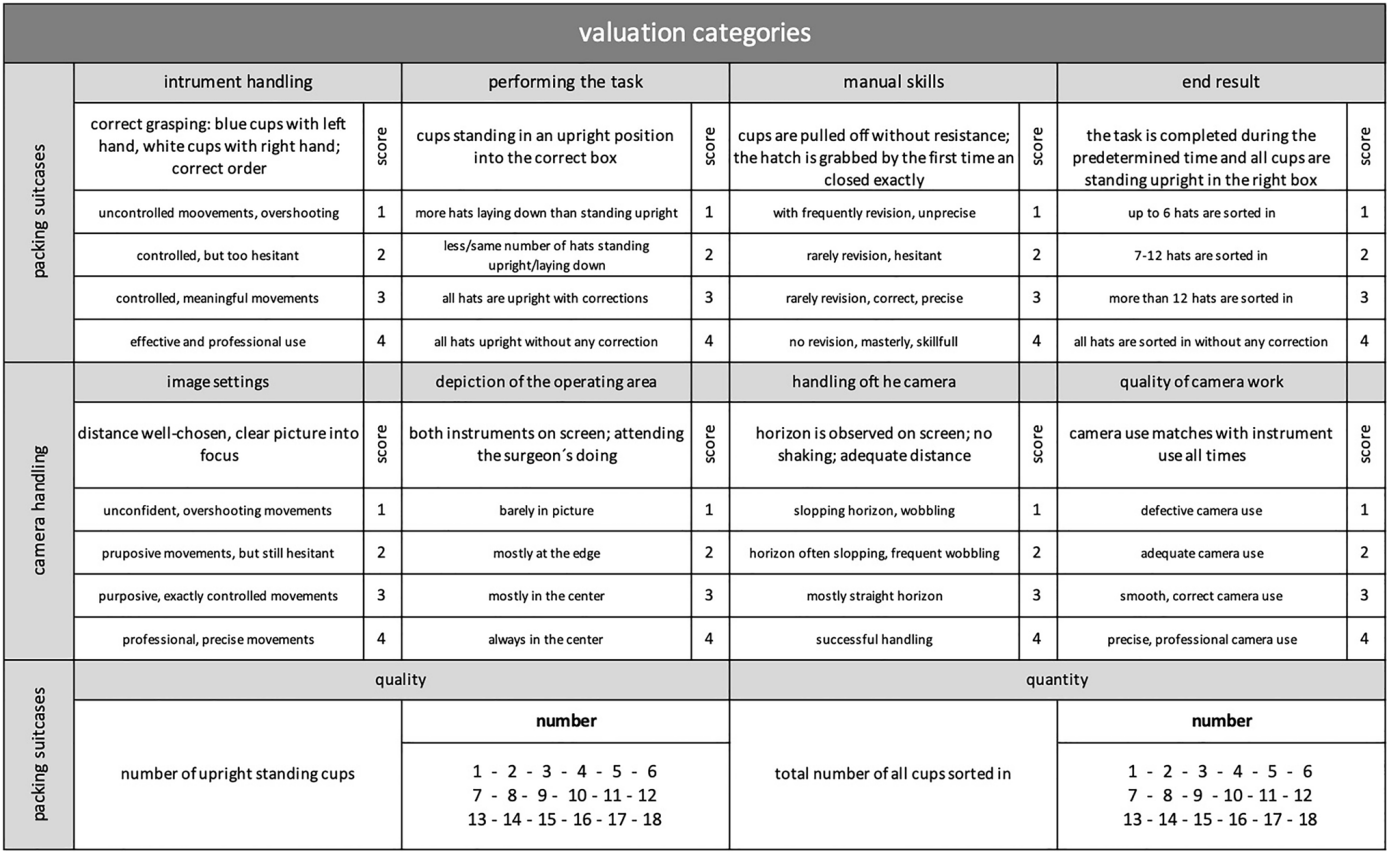
The checklist CAT to assess the laparoscopic performance

Two blinded raters evaluated the pseudonymized assessment videos using a validated evaluation sheet. The assessment checklist “competency assessment tool” (CAT) was adapted for the purposes of the present study [32] (Fig. 2). The quality of performance was defined as the number of upright cups and the quantity of performance was defined as the total number of cups positioned in the correct case regardless of whether they stand upright.

### Statistics

Statistical analysis was performed using IBM SPSS 25.0, 26.0 and 27.0 (IBM SPSS, Armonk, New York, USA), R 3.6.3 (R: A language and environment for statistical computing, R Foundation for Statistical Computing, Vienna, Austria) and Mplus 7 (www.statmodel.com). Significance was set at p < 0.05. Descriptive analyses consisted of mean (MV), median, minimum (min) and maximum (max) values as well as the standard deviation (SD).

Inter-rater reliability was described by the extended percentage agreement method [33]. An agreement of 100% and a tolerance of one scale point indicated that both examiners differed by a maximum of one point for each item assessed. Reliability was calculated using the Finn coefficient, which varies between 0 and 1 [34]. A value > 0.7 was considered as good [34].

Inferential statistical analysis consisted of four steps:

1. The performance regardless of group membership was investigated with a repeated measure ANOVA.
2. Performance depending on group membership was calculated using Welch's test as well as single factor variance and analysis of covariance or as repeated measure ANOVA.
3. Factor analysis was performed to inspect whether individual questionnaire items could be combined to a scale. Bartletts test and the Kaiser–Meyer–Olkin coefficient (KMO coefficient) were employed to assess whether the data was suited for a factor analysis.
4. A latent difference model was specified to summarize the interplay of steps 1–3 [35]. A latent difference model is a structural equation modeling technique. It has been developed to conduct latent longitudinal analyses free of measurement error and is mathematically superior to results which are calculated on the manifest level. Usual fit indices (e.g. Comparative Fit Index, threshold = 0.95) were employed to assess the quality of the model.

## Results

### Sample

One hundred and forty-three students participated in the study. Of these, 17 (11.9%) were excluded from further analysis due to insufficient video footage, missing questionnaire entries, or drop-out.

The IG comprised 58 participants (MV: 25.8 years, female: 63.8%) and the CG 68 participants (MV: 26.0 years, female: 64.7%). The IG and CG did not differ regarding gender, age, prior laparoscopic experience and self-assessed motivation (Table 1).

**Table 1:** Characteristics of IG and CG

		IG		CG		
		total	SD	total	SD	*p*-value^*2^
Participants (n)		58		68		
Gender (f/m)		37/21		44/24		0.94^*3^
Age (years)		25.78	± 2.39	26.04	± 2.79	0.31^*4^
Prior laparoscopic experience (mean)		1.72^*5^	± 0.62	1.64^*5^	± 0.62	0.22^*4^
Motivation (mean)		3.38^*5^	± 1.0	3.21^*5^	± 1.03	0.10^*4^

^*^^1^standard deviation *^2^group-differencese*,* *^3^chi-quadrat-test with Yates ‘continuity correction, *^4^ welch-test, *^5^ mean value of the entry questionnaire

### Questionnaire evaluation and factor analysis

Only 7% of participants reported prior laparoscopic experience, e.g. during clerkships. Overall, participants were highly motivated (MV: 3.38, on a five-point-Likert-scale). Barlett’s test (*p* < 0.001) and KMO (0.82) confirmed suitability of the data for factor analysis and a one factor solution (self-assessed dexterity) was favored. Five items had factor loadings > 0.30 and Cronbach's alpha as a measure of internal consistency exceeded 0.6 including “manual skills”, “fine motor skills”, “steady hand”, “use manual adroitness”, and “eye-hand-coordination”.

### Internal validation and inter-rater reliability

**Fig. 3:**
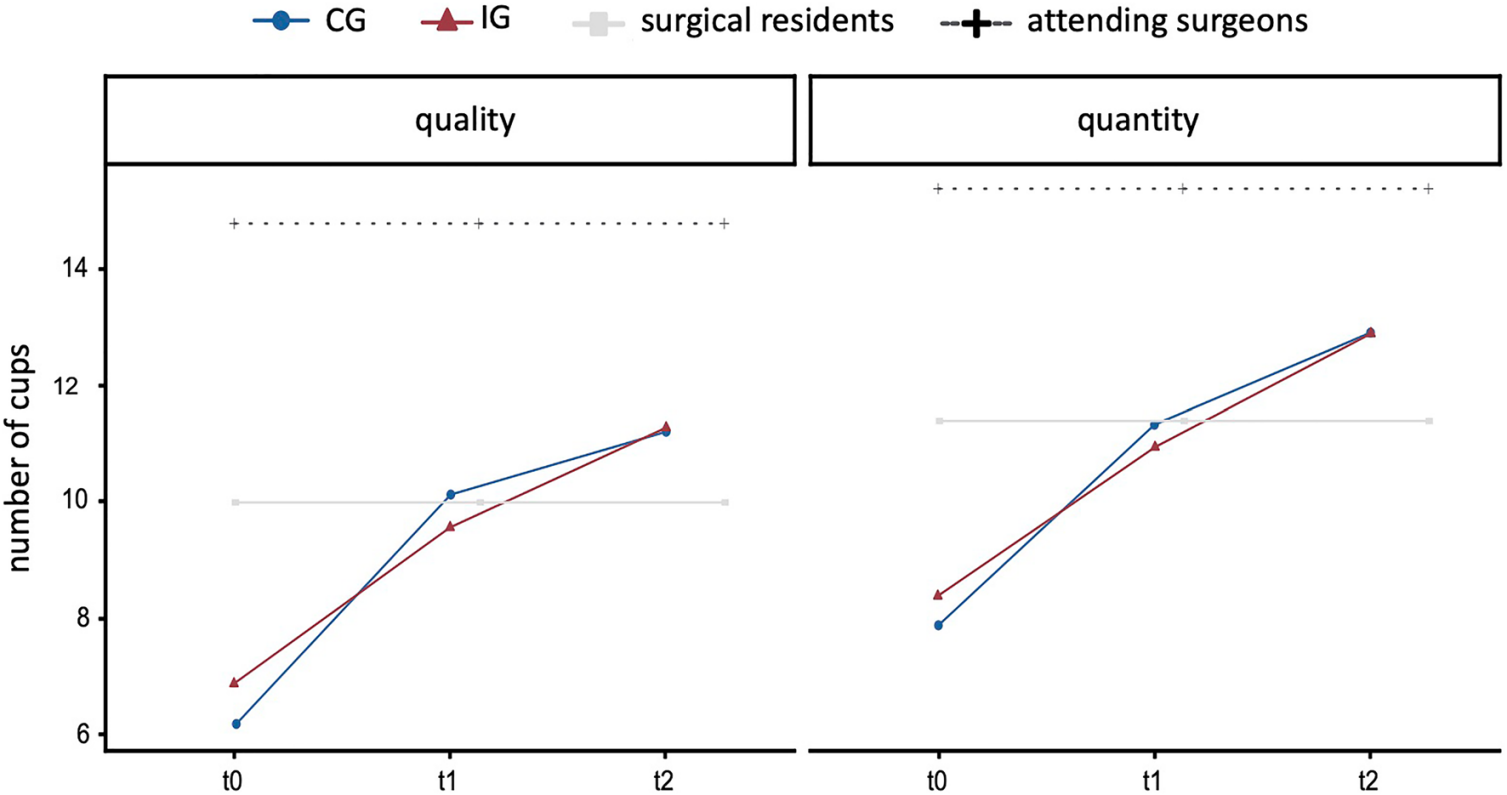
Internal validation. Increase in performance of students in the control group (CG) and intervention group (IG) compared to surgical residents and attending surgeons regarding the categories “quality” and “quantity”

Attending surgeons and surgical residents performed “packing suitcases” solely at one time point. We used their performance as a gold standard for comparison with less experienced users. As expected, attending surgeons outperformed students in all categories (*p* < 0.05). Similarly, surgical residents showed significantly better performance than students (*p* < 0.05) in the three assessment categories of the CAT: "instrument handling", "manual skills" and "end result" (data not shown). Throughout the course, participants improved their laparoscopic skills so that their final assessment (t_2_) was comparable to the surgical residents in “quality” and “quantity” (Fig. 3).

Extended percentage agreement was 97.2% with a tolerance of 1 scale point. The Finn coefficient was 0.72 indicating high inter-rater reliability.

### Increase in performance independent of group membership

All participants increased their laparoscopic performance (in role of the surgeon and camera-assistant from t_0_ to the final assessment at the end of the course (t_2_). The effect was independent of whether participants belonged to the IG or CG (Table 2).

**Table 2:** Performance of participants independent of group membership from t_0_ to t_2_

		t_0_		t_2_		
		MV	SD	MV	SD	*p-value*
Packing suitcases						
Instrument handling		1.98	± 0.67	2.86	± 0.74	** < 0.001**
Performing the task		2.37	± 0.86	2.51	± 0.79	Not significant (n.s.)
Manual skills		1.87	± 0.69	2.53	± 0.82	** < 0.001**
End result		1.85	± 0.46	2.56	± 0.57	** < 0.001**
Camera handling						
Image settings		2.59	± 0.60	2.79	± 0.69	** < 0.05**
Depiction of the operating area		2.56	± 0.93	2.63	± 0.76	n.s
Handling of the camera		2.13	± 0.76	2.30	± 0.68	** < 0.05**
Quality of camera handling		2.44	± 0.75	2.75	± 0.79	** < 0.001**
Number of cups						
Quality		6.49*	± 2.76	11.26*	± 3.18	** < 0.001**
Quantity		8.11*	± 2.29	12.91*	± 2.63	** < 0.001**

^*^Number of cups; significance (p-value) is defined as the difference between t_0_ to t_2_

### Increase in performance depending on group membership

**Fig. 4:**
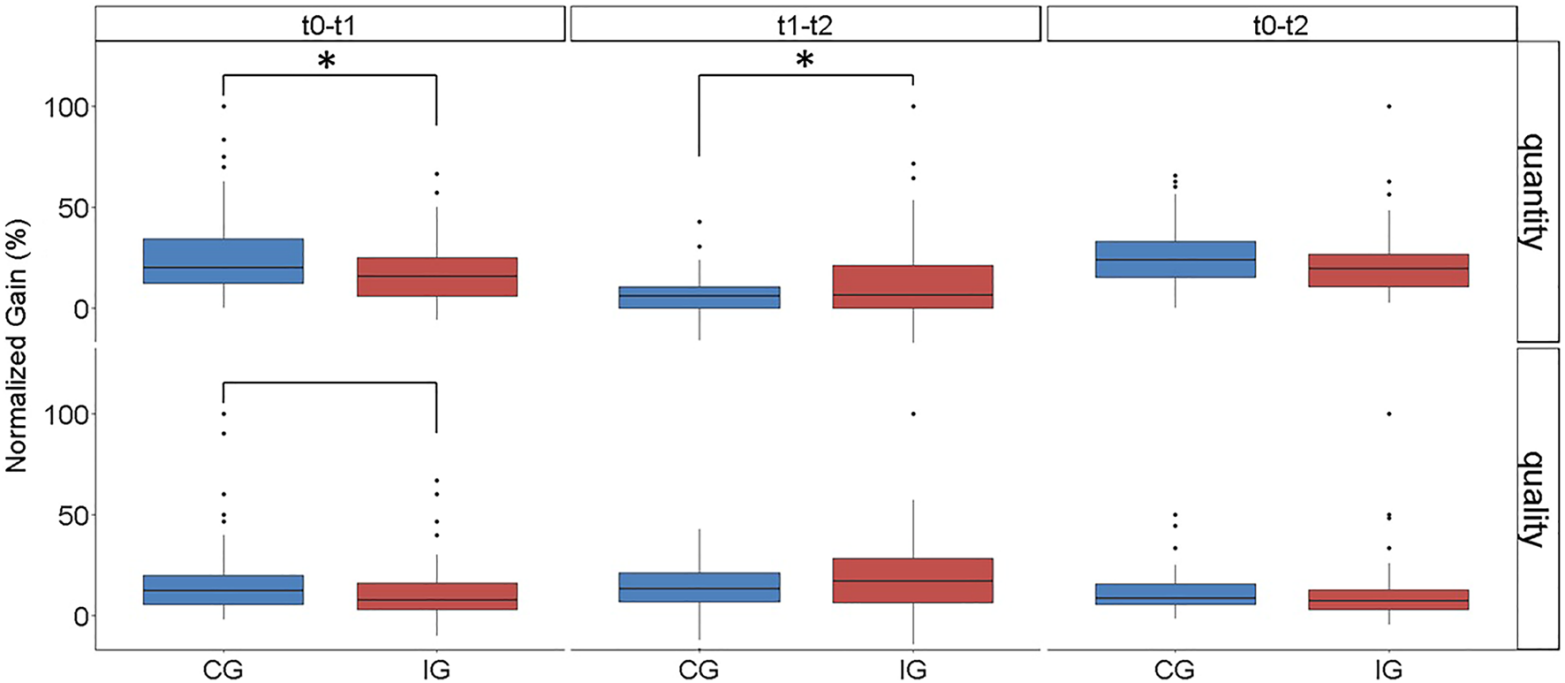
Increase in performance given as the normalized gain of “quality” and “quantity” *p-value ≤ 0,05

Overall the performance from baseline (t_0_) to final assessment (t_2_) did not differ. From t_0 _to t_1,_ however the CG experienced a significantly higher performance increase (“quantity” and “quality”) compared to the IG, whereas the IG showed a significantly higher increase in performance from t_1 _to t_2_ (quantity) (Fig. 4).

### Influence of covariates

**Fig. 5:**
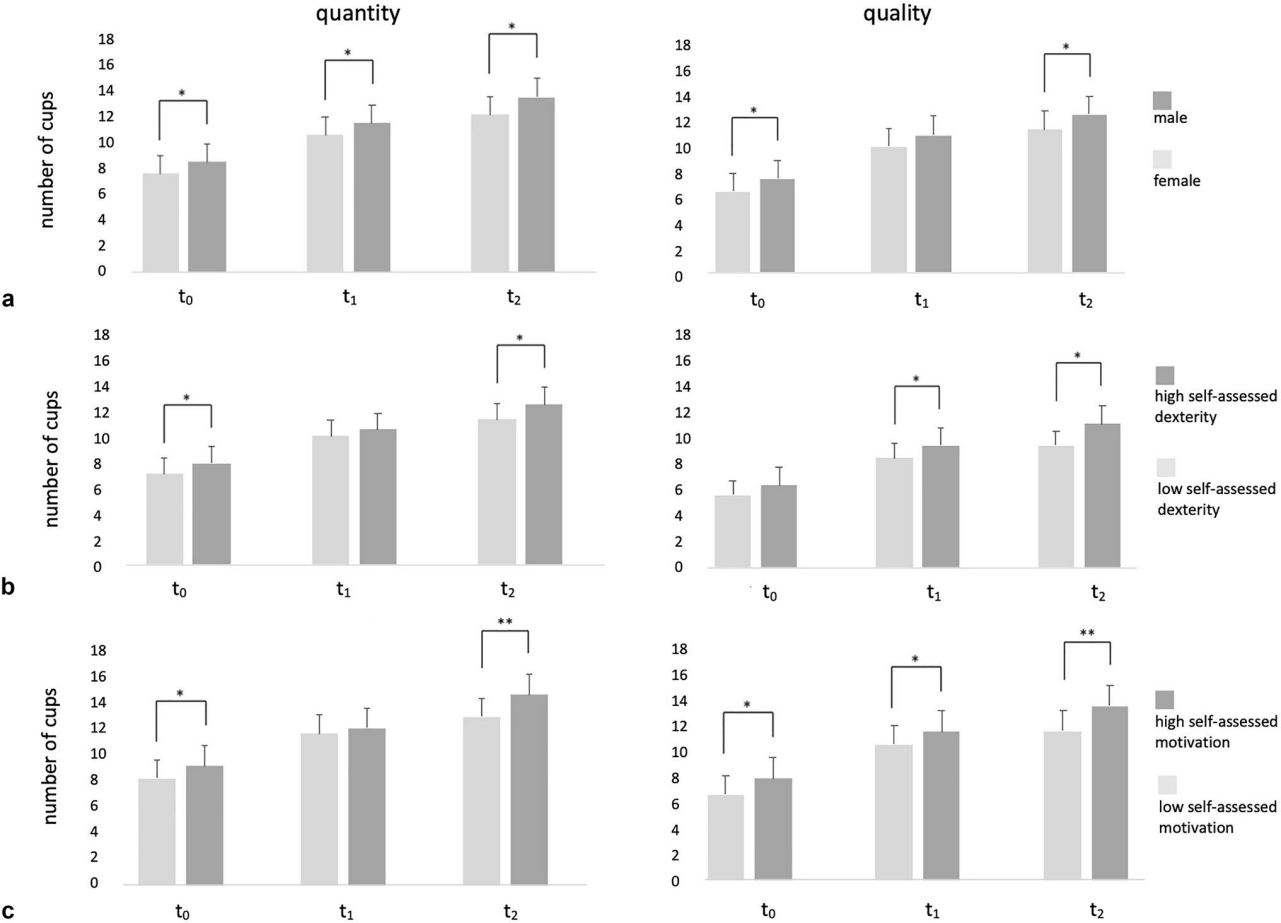
Quantitative and qualitative performance at t_0_, t_1_ and t_2_ by **a** gender, **b** self-assessed dexterity and **c** self-assessed motivation, *p ≤ 0,05; **p ≤ 0,001

The impact of potential covariates on “quality” and “quantity” was assessed for gender, self-assessed dexterity and self-assessed motivation (Fig. 5).

#### Gender

Gender had an impact on performance. Female participants correctly placed significantly (*p* < 0.05) less cups in the right case (“quantity”). Additionally, female participants showed significantly (*p* < 0.05) lower quality at two time-points (t_0_: 6.14 cups and t_2_: 10.85 cups) compared to their male counterparts (t_0_: 7.14 cups and t_2_: 12.00 cups).

#### Dexterity

Self-assessed dexterity was classified according to the calculated percentiles as "low" (0.00 to 2.60) and as "high" (3.31 to 5.00). Students, who rated themselves with high manual skills, showed better performance regarding quality from time-point t_1 _to t_2_. At baseline (t_0_) and the final assessment (t_2_), they also achieved higher scores regarding quantity.

#### Motivation

Self-assessed motivation was subdivided into two groups "low" and "high", based on the results of the question "I am more motivated than average on this course". This partition was made at MV 3.0 since percentile ranges were unequal. At each time point, there was a significant difference between the two groups of students. Participants who were highly motivated achieved higher quality at all time-points as well as a higher quantity at baseline (t_0_) and final assessment (t_2_).

#### Latent difference model

**Fig. 6:**
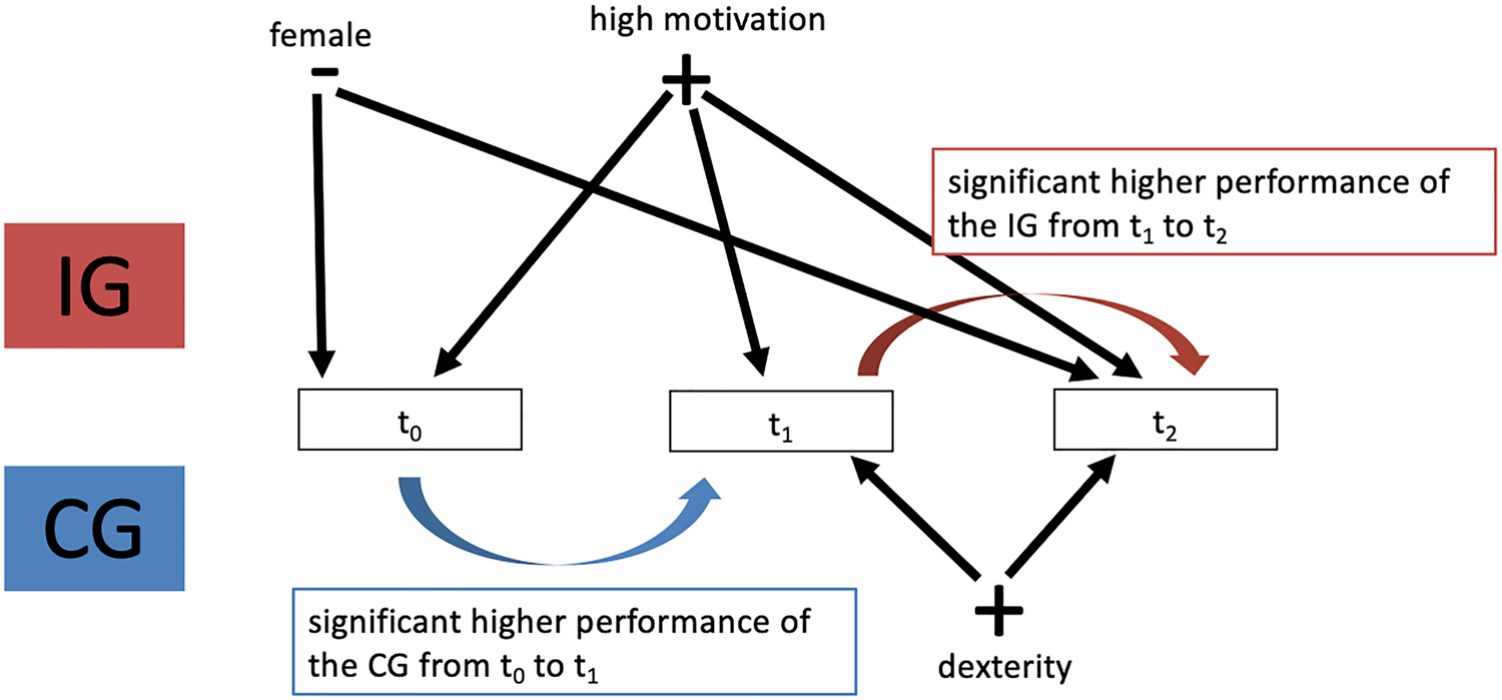
Simplified version of a latent difference model

A latent-difference model was used to demonstrate the relationship of the covariates, the IG and CG on performance at each time-point. The closer Lambda (λ) approaches “1” the higher the dependency between two variables. A simplified version of the latent difference model is shown in Fig. 6. To simplify the presentation, only the influence on quality (upright standing cups) is shown in the figure.

For the covariate gender, the loading λ=0.953 was associated with significantly higher baseline performance (*p* < 0.05). Self-assessed motivation of the participants had a significant influence on the baseline results with λ = 1.125. Self-assessed motivation also impacted improvements in performance at time point t_1 _(λ = 0.732) and t_2 _(λ = 0.855). The CG showed a significantly higher increase in performance from t_0 _to t_1_. The IG showed a higher increase in performance from t_1 _to t_2_.

## Discussion

### Laparoscopic skill training

In this prospective randomized study, performance during a standardized laparoscopy course was evaluated with a focus on learning activity and potential influencing factors. In line with other studies [17, 36, 37], all participants showed a significant increase in performance throughout the course [17, 38]. Although not examined in the present study, research has shown the likelihood of laparoscopic skill transfer from training to the clinical setting [16, 39, 40] in particular the LTB technique [31, 41]. Here, a modified version integrating a mental exercise was developed and employed [33, 34]. Although a positive effect of mental exercises on learning efficiency is widely acknowledged [42], little attention has been paid to its implementation in laparoscopic training courses [43, 44].

### Different proficiency levels prove internal validity

Validity of the module “packing suitcases” was investigated by analyzing performance of attending surgeons and surgical residents. As expected, attending surgeons outperformed surgical residents, who in turn were superior to laparoscopy-naïve participants at baseline. Similarly, Hassan et al. showed that different levels of competence, from professional to novice, could be differentiated when analyzing performance on a laparoscopic trainer [45]

### Deconstruction into key steps

The present study evaluated whether the teaching method DIKS could decrease the required time to practice. A significant and continuous improvement in performance from baseline (t_0_) to the end of the course (t_2_) was found, independent of the teaching method. Overall, there were no significant differences between the two groups over the entire study period (t_0 _t_1_ t_2_). However, distinct differences in performance depending on the teaching method (IG or CG) were found to be associated with the time point of training. Increased time to practice led to a more pronounced early increase in performance (t_0 _to t_1_, p < 0.05), whereas DIKS exerted its beneficial effect on prolonged learning (t_1 _to t_2_), while practical training time was reduced by 58%.

These results confirm that at the beginning of learning a new practical skill, adequate time to practice (manual handling of instruments and cameras) results in an immediate improvement in performance. Additional mental exercises may compensate for significantly reduced time to practice. This finding is in line with previous studies which found that mental exercise in surgical training can have a positive effect on learning success and is a cost- and time-efficient strategy [42, 46–49]. One study even demonstrated that additional mental exercise such as DIKS may lead to superior results compared to practical exercise [42]. This contrasts with the concept of “see one, do one, teach one …”, which is widely used in surgery [50].

### Covariates

The participants' self-assessment of dexterity significantly impacted performance. Specifically, those who rated themselves poorly showed significantly worse performance than students who were convinced of their motor skills [51].

A significant positive correlation between high self-assessed motivation and performance was also found throughout this study [52]. The extent to which teaching methods influence self-assessed motivation in medical education requires further research.

Unexpectedly, we found that gender influenced performance with male participants significantly outperforming female participants. This might be because females tend to be more concerned about making mistakes, and subsequently require greater time to complete a given task [53]. In this context, males seem to benefit from their tendency to take risks and be more self-confident [53, 54]. The difference between genders in surgical training is in line with the systematic review of Ali et al. which included 247 studies [55, 56].

Contrary to our hypothesis, camera handling did not impact operative outcomes [57]. This may be due to a methodological artifact: Surgeon and camera-assistant formed a permanent team throughout the course. Future investigations would need to include both weak and strong camera assistance with an experienced as well as an inexperienced surgeon.

### Latent difference model

After analyzing all covariates, a latent-difference model was implemented to investigate inter-individual differences on a measurement-free level [58]. This model is mathematically sound in studies that consider changes over a certain period of time [58]. As expected, the latent-difference model prevented distortions and increased the informative value of the data.

### Strengths and limitations

The prospective randomized design and sample size are strengths of the study. The high number of participants enabled differentiated subgroup and covariate analysis. Similar studies had significantly less participants [59, 60]. Since the study was conducted during a curricular surgical internship, highly as well as less motivated participants took part avoiding selection bias. Furthermore, the standardized setting of the course with supervision and standardized instructional videos ensured comparable settings. The performance was evaluated using a standardized evaluation sheet and a behaviorally anchored rating scale, which has been described and evaluated earlier [61].

### Conclusions

We showed that novices were able to significantly improve their skills during a laparoscopic surgical course. DIKS compensated for shorter practice time and thereby proved to be a valuable tool to optimize performance outcome. Covariates such as gender, self-assessed motivation and self-assessed dexterity significantly influenced training outcome emphasizing the importance of tailored training interventions.

The aim of the study was to emphasize the importance of efficient structured laparoscopic training combined with sound psychological learning techniques. `DIKS` can be easily integrated into the daily work routine. Regardless of the career stage, complex motion sequences require elaborated educational techniques based on learning and instructional psychology. Much more research in the field of medical education needs to explore, validate and disseminate knowledge on learning and teaching professional practical skills.
